# Uterine inflammatory myofibroblastic tumor: more common than expected: Case report and review

**DOI:** 10.1097/MD.0000000000008974

**Published:** 2017-12-01

**Authors:** Vincenzo Dario Mandato, Riccardo Valli, Valentina Mastrofilippo, Alessandra Bisagni, Lorenzo Aguzzoli, Giovanni Battista La Sala

**Affiliations:** a Unit of Obstetrics and Gynecology; b Unit of Pathology; c Unit of Surgical Gynecologic Oncology, Azienda Unità Sanitaria Locale-IRCCS di Reggio Emilia; d Unit of Obstetrics and Gynecology, University of Modena e Reggio Emilia, Reggio Emilia, Italy.

**Keywords:** anaplastic lymphoma kinase, diagnosis, laparoscopy, treatment, uterine inflammatory myofibroblastic tumor

## Abstract

**Rationale:**

Inflammatory myofibroblastic tumor (IMT) is a rare mesenchymal neoplasm composed of spindled to epithelioid cells with prominent myxoid stroma and inflammatory infiltrate. It has a low but definite malignant potential. However, its management has never been standardized.

**Patient Concerns and Diagnosis:**

We present the first case of uterine IMT laparoscopically treated. Moreover, we reviewed the English literature regarding uterine IMT published between 1987 and June 2017. A total of 72 cases of uterine IMT were included. Clinical and pathological characteristics, treatments and outcomes were recorded.

**Interventions and Outcomes:**

A total laparoscopic hysterectomy with opportunistic bilateral salpingectomy was performed. Patient is free of disease at 6 months of follow-up.

**Lessons:**

Uterine IMT may be identified by anaplastic lymphoma kinase overexpression, its prognosis is usually good, complete excision seems to be effective to avoid relapse and mini invasive surgery seems to be effective and safe to treat uterine IMT. However, considering the age of women affected by disease, conservative management, or medical therapy could be taken in account to avoid surgical injuries and to preserve fertility.

## 1 Introduction

The first case of inflammatory myofibroblastic tumor (IMT) has been described in the lung in the 1973.^[1]^ Originally IMT has been included in the heterogeneous category of inflammatory pseudotumor.^[2]^ Subsequently, IMT was better characterized by molecular^[3]^ and subsequent genetic tests^[4]^ and according to its biological behavior.^[5]^ Nowadays it represents a distinct neoplastic process.^[6]^ IMT is a mesenchymal neoplasm of low but definite malignant potential that is composed of a population of spindled to epithelioid cells set in a myxoid stroma, usually associated with a conspicuous lymphoplasmacytic infiltrate.^[7,8]^ Approximately 50% of IMT presents a genetic rearrangement of anaplastic lymphoma kinase (ALK) gene located in the chromosomal region 2p23.^[8–12]^ This rearrangement is more frequent in children and young adults.^[6]^ Generally, IMT may arise in multiple organs, most commonly lungs, mesentery, omentum, and retroperitoneum.^[8]^ Rarely IMT arise in the uterus. Since the first case described by Gilks et al in the 1987,^[9]^ 72 cases of uterine IMT has been reported in literature.^[3–26,27]^ Here, we describe the first case of laparoscopy treated uterine IMT and review all cases of IMT arising from the uterus since 1987 to report the most useful diagnostic criteria, to identify the best treatment options and to clarify the outcome of this disease.

## 2 Case report

**Figure 1:**
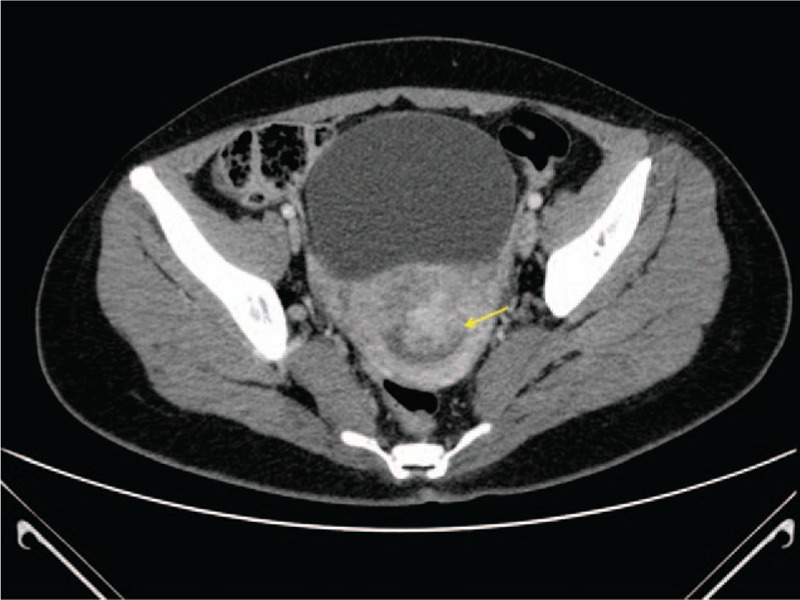
Computed tomography scan showing an intrauterine mass (yellow arrow).

**Figure 2:**
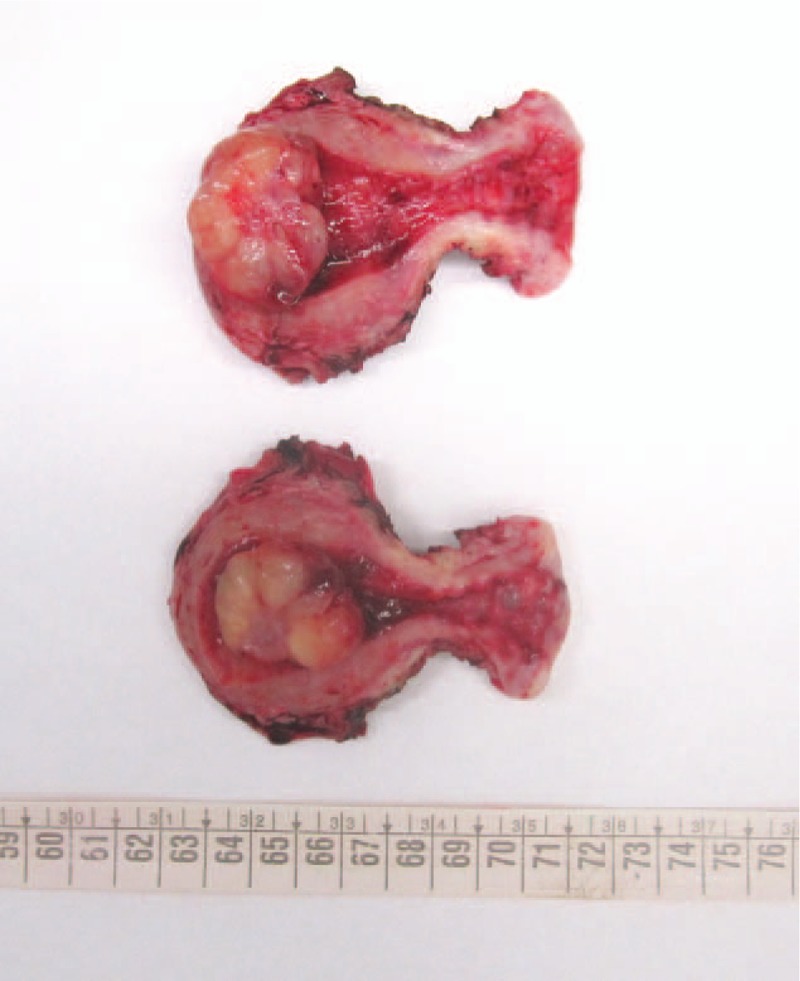
The gross appearance of the hysterectomy specimen. A polypoid lesion widens the endometrial cavity. The lesion is macroscopically glistening, with a narrow stalk.

**Figure 3:**
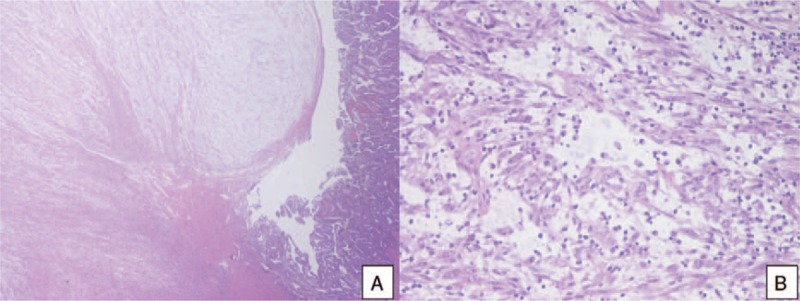
A, A low power view of the polypoid lesion (left) and the adjacent endometrial mucosa (right) (hematoxylin-eosin, 2×). B, At high power, the polypoid lesion consisted of a proliferation of spindle, myoid cells, with mild atypia, set in a myxoid, lymphocyte-rich stroma. (hematoxylin-eosin, 20×).

**Figure 4:**
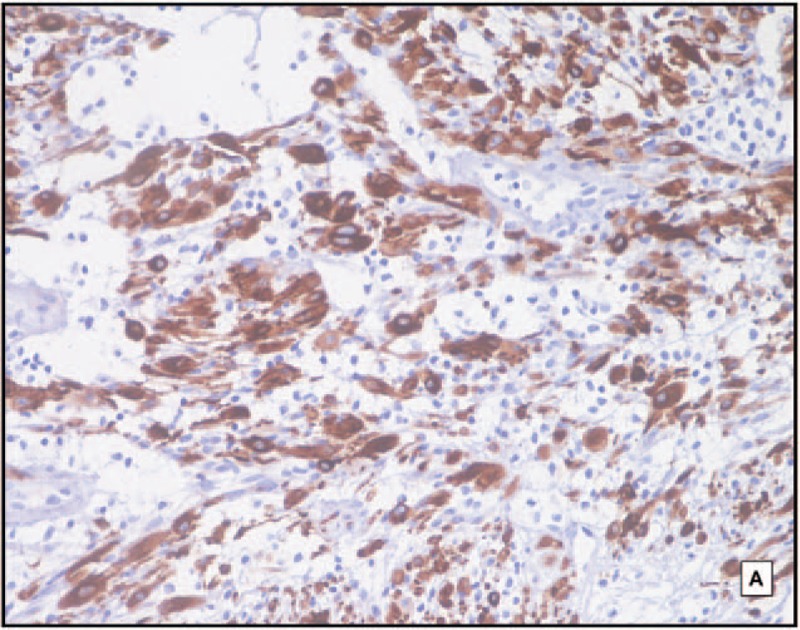
Immunohistochemical stains show the following results: strong cytoplasmic positivity for anaplastic lymphoma kinase (ALK) (20×, hematoxylin counterstain).

**Figure 5:**
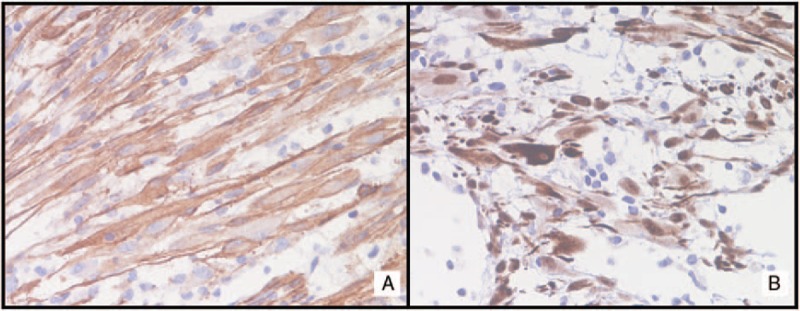
Immunohistochemical stains show the following results. A, Diffuse positivity for smooth muscle actin (40×, hematoxylin counterstain). B, Strong nuclear stain for WT1 (40×, hematoxylin counterstain).

**Figure 6:**
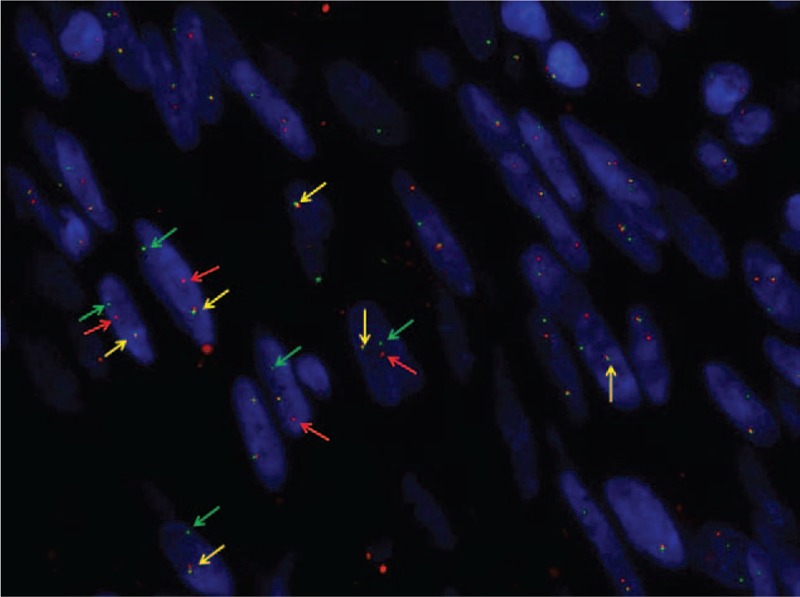
The anaplastic lymphoma kinase (ALK) rearranged positive cells presented one fused signal (yellow arrows) with single green (green arrows) and single orange (red arrows) signal.

A 36-year-old woman was referred to the Department of Obstetrics and Gynecology with a severe vaginal bleeding. Hemoglobin level decreased from 12.1 to 10.2 g/dL. Her history included 2 vaginal deliveries, but was otherwise unremarkable. Gynecological evaluation and transvaginal ultrasound found a huge intrauterine mass coming out through the cervix. A diagnostic hysteroscopy with biopsy of the mass was performed. At histological examination an IMT of the uterus was diagnosed. A computed tomography scan of the abdomen and pelvis confirmed the intrauterine extension of the IMT and excluded myometrial infiltration, extrauterine involvement or metastatic spread (Fig. 1). Considering that the patient had a persistent vaginal bleeding and did not want fertility preservation, we performed a total laparoscopic hysterectomy with opportunistic bilateral salpingectomy. Pathological examination was performed. Grossly, the uterine cavity was occupied by a polypoid lesion, measuring 3 cm across without myometrial infiltration (Fig. 2). Histologically, the neoplasia was composed of plump spindle cells, with vescicular nuclei showing small eosinophylic nucleoli. The neoplastic cells were set in a myxoid, inflammatory background (Fig. 3). There was no necrosis; the mitotic activity was low, with only 1 mitosis per 10 high power fields (HPFs). At immunohistochemistry, the neoplastic cells were positive for ALK (Fig. 4) (cytoplasmic positivity), smooth muscle actin, WT1 (Fig. 5), and (focally) pancytokeratin, whereas they were negative for CD117, CD10, and p53 (Fig. 4). Fluorescent in situ hybridization (FISH) test for ALK gene rearrangement (with break-apart probe) was positive (Fig. 6). In fact, neoplastic cells showed ALK rearrangement in 91% of cell nuclei. Most of the cells had classical positive pattern signals: the cells showed coexistence of 1 fused signal with 2 single orange and green signals (1O1G1F). The diagnosis of IMT of the uterus was confirmed. Our patient is free of disease (FOD) at 6 months of follow-up.

## 3 Materials and methods

### 3.1 Ethical review

Ethical approval was not necessary in case of case report publication and patient gave her informed consent to collect data and images for publication.

### 3.2 Immunohistochemistry

The following primary monoclonal antibodies were used: ALK (clone ALK01, prediluted; Ventana/Roche), smooth muscle actin (clone 1A4, prediluted; Ventana/Roche), WT1 (clone 6F-H2, prediluted; Ventana/Roche), p53 (clone DO-7, prediluted; Ventana/Roche), pancytokeratin (clone AE1+AE3+PCK26, prediluted; Ventana/Roche), CD10 (clone SP67, prediluted; Ventana/Roche), and CD117 (polyclonal, dilution 1/200; Dako/Agilent). Four-micrometer-thick sections on silane-coated slides were stained using the Benchmark XT immunostainer (Ventana/Roche, Tucson, AZ).

### 3.3 Fluorescence in situ hybridization

A representative formalin-fixed, paraffin-embedded tissue block was selected for FISH. Four-micrometer-thick sections were incubated overnight at 56°C. Deparaffinization, pretreatment, enzyme digestion, and fixation of slide were performed using the Vysis paraffin pretreatment kit (Abbott Molecular, Des Plaines, IL), according to the manufacturer's recommended protocol. Denaturation and hybridization were performed in a ThermoBrite denaturation/hybridization system for FISH (Abbott Molecular). Five microliters of ALK probe (Vysis LSIALK Break Apart FISH Probes; Abbott Park, IL) were applied to the tissue section and then it was denatured at 85°C for 1 minute and hybridized overnight at 37°C. Then slide was washed in wash buffer at 72°C for 4 minutes and counterstained with 10 μL 4’-6’diamidino-2-phenilindole). FISH images were analyzed with a Leyca DM5500 fluorescence microscope (Leyca Biosystem). The negative pattern is represented by 2 fusion signal or by 1 fusion signal and an isolated green signal. The positive classical pattern is represented by 1 fusion signal and 2 separated orange and green signals. The other positive pattern is represented by 2 or more separate orange and green signals or by 1 fusion signal and 1 isolated orange signal without the corresponding green signal. Green and orange signals must be separated by more than twice the size of an isolated signal.

### 3.4 Systematic review of the literature

We collected and analyzed articles published on IMT between 1987 and Jun 2017 using PubMed as a database and the following search terms: “inflammatory myofibroblastic tumor and uterus,” “inflammatory myofibroblastic tumor and ALK,” “inflammatory myofibroblastic tumor and cervix,” “uterine inflammatory myofibroblastic tumor,” and “inflammatory myofibroblastic tumor and female genital tract.” After selecting for cases arising from uterus, 72 reports of IMT were found (Table 1  ). Particularly, we recorded histological, immunohistochemical and genetic findings, treatment, and outcomes of the IMTs described in literature. Overall survival (OS) was computed as the time period from the date of treatment to either the date of death or last follow-up. Disease-free survival (DFS) was computed as the disease-free period from the date of treatment to the date of relapse or last follow-up.

**Table 1:**
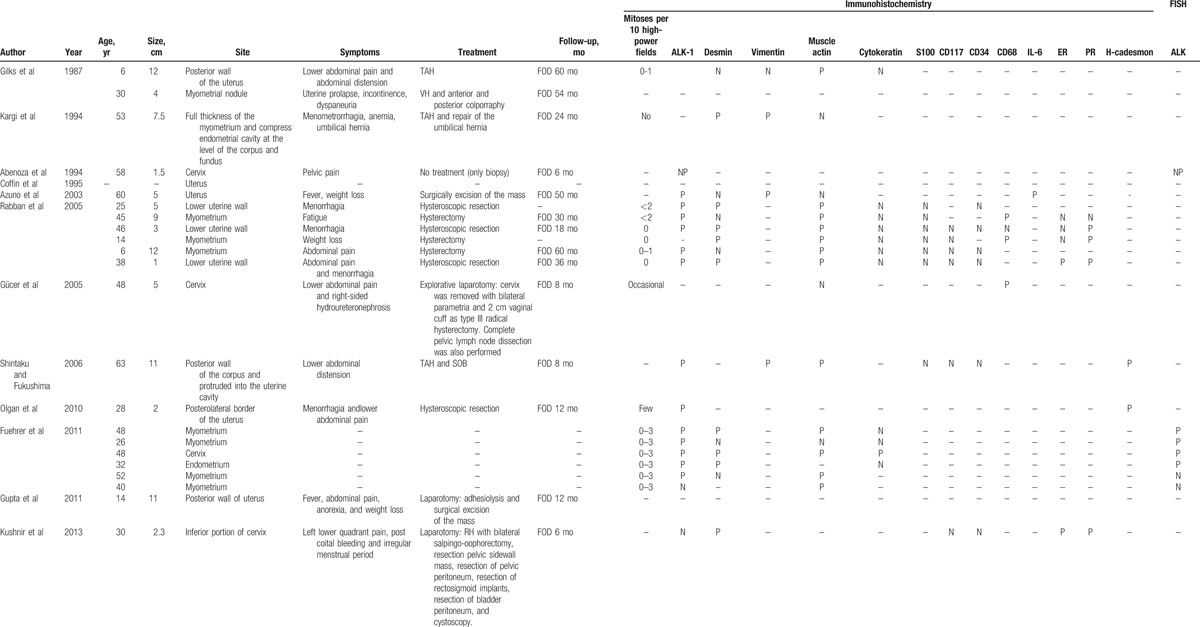
Clinical and pathological features of 72 patients with inflammatory myofibroblastic tumor reported in literature.

**Table 1 (Continued):**
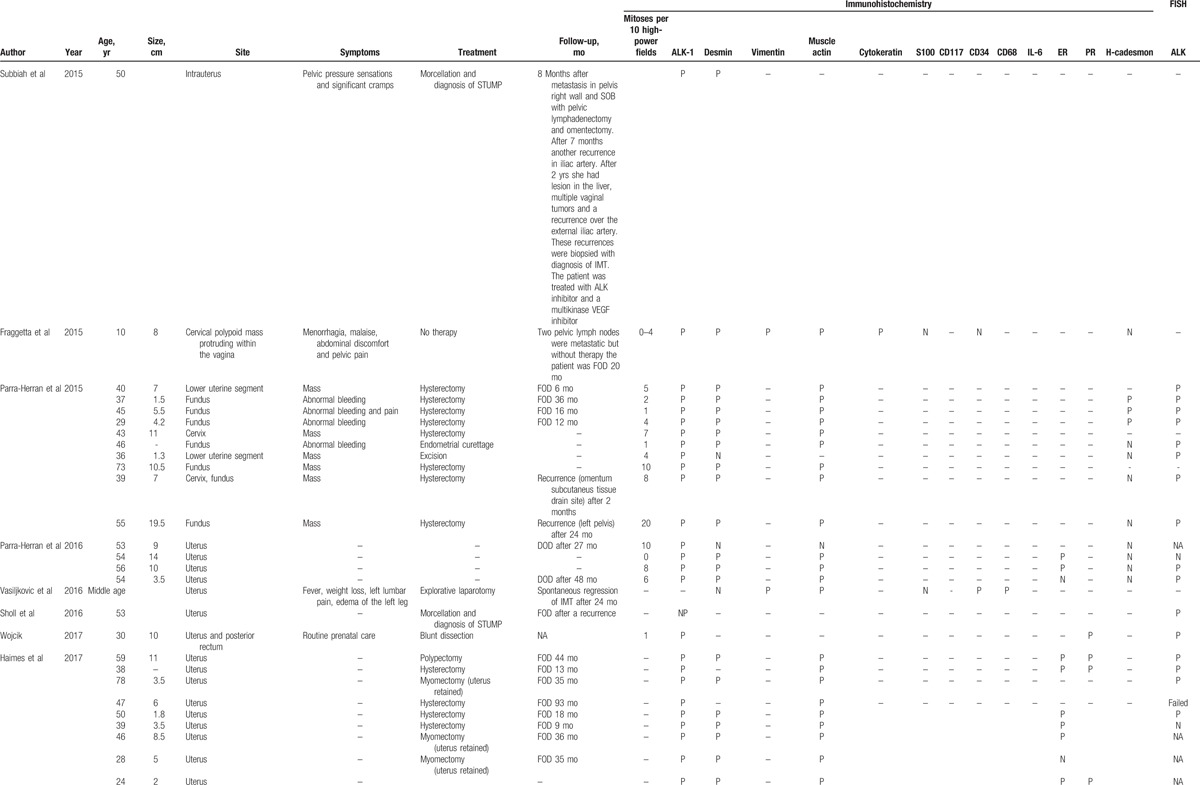
Clinical and pathological features of 72 patients with inflammatory myofibroblastic tumor reported in literature.

**Table 1 (Continued):**
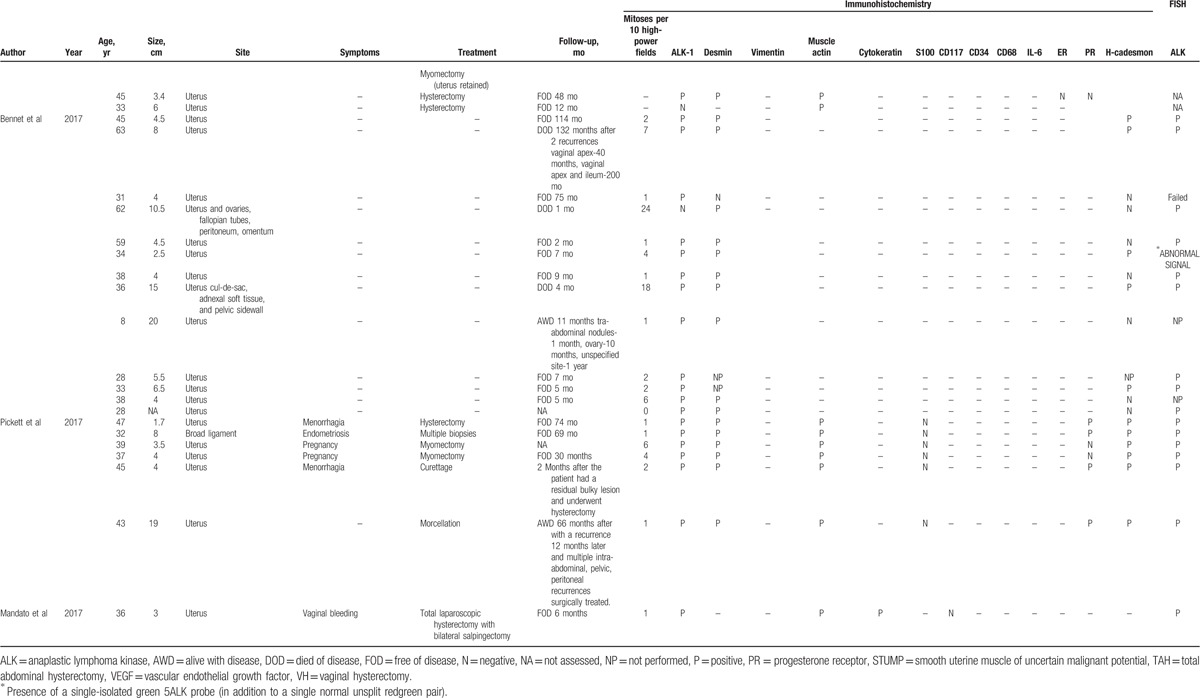
Clinical and pathological features of 72 patients with inflammatory myofibroblastic tumor reported in literature.

## 4 Review results

### 4.1 Clinical features

Table 1   shows the main clinical features of all 72 IMT cases reported in literature. In 1 case^[15]^ no information other than the site of origin was available. The age at presentation of the 72 patients ranged from 6 to 78 years (mean, 40.6 ± 14.9 years). Overall, 65 of 72 (90.3%) patients presented with an IMT arising from the uterine corpus and 7 of 72 (9.7%) presented with an IMT arising from the uterine cervix. Information on tumor size was available for 58 of 72 (80.5%) patients. The size ranged from 1 to 20 cm with a mean size of 6.8 cm [standard deviation (SD) ± 4.4 cm]. Symptoms were reported for 35 of 72 (48.6%) patients. Abdominal/pelvic pain was reported for 12 of 35 (34.3%) patients, vaginal bleeding for 13 of 35 (37.1%), fever/weight loss for 5 of 35 (14.3%), abdominal distension for 3 of 35 (8.6%), urinary disorders for 2 of 35 (5.7%), fatigue for 2 of 35 (5.7%), uterine prolapse for 1 of 35 (2.9%), 6 of 35 (17.1%) patients complained about the appearance of a mass, 3 of 35 (8.6%) pregnant woman discovered the IMT during prenatal routine visit, and 1 of 35 (2.9%) patient discovered the IMT during surgery for endometriosis. Information about management after diagnosis was reported for 48 of 72 (66.7%) patients. Only follow-up without treatment was reported for 4 of 48 (8.3%) patients,^[20–22,26,27]^ surgery was reported for 44 of 48 (91.7%) patients.^[3,6,12,14,17–19,23,24,26,27]^ Of the 44 patients who received surgical treatment, 23 (52.3%) underwent hysterectomy,^[6–9,12,14,18,26,27]^ 4/44 (9.1%) underwent hysteroscopic resection,^[8,10]^ 10 (22.7%) underwent tumorectomy,^[3,6,7,17,24,26,27]^ 1 (2.3%) underwent radical trachelectomy with pelvic lymph-adenectomy,^[16]^ 3 (6.8%) underwent morcellation,^[19,23,26,27]^ 2 of 44 (4.5%) underwent endometrial curettage,^[6,26,27]^ and 1 of 44 (2.3%) underwent polypectomy.^[7]^

### 4.2 Follow-up data

Follow-up information was available for 53 of 72 (73.6%) patients (Table 1  ). Out of 53 patients 46 (86.8%) patients were FOD despite having one or more relapses during follow-up and 5 (9.4%) patients died of disease (DOD) at 1, 4, 27, 48, and 132^[25]^ months after treatment.^[13]^ Mean OS in the 46 FOD patients was 29.2 months. Out of 46 (71.7%) FOD patients 33 received surgical treatment, and their mean follow-up was of 29.3 months with a median OS of 24 months. Out of 46 (8.7%) FOD patients 4 were only followed-up with spontaneous regression of IMT, and their mean follow-up period was of 29.7 months and the median OS was of 22 months and for 9 of 46 (19.6%) patients we have no information about the treatment. Out of 53 (15.1%) patients 8 had recurrences.^[6,19,23,25,26,27]^ 5 of 8 (62.5%) were FOD,^[6,19,23,26,27]^ particularly, 1 of 5 (20%) patient had a residual bulky lesion and underwent hysterectomy after 2 months,^[26,27]^ 1 of 5 (20%) patient had omental recurrence 2 months after treatment,^[6]^ 1 of 5 (20%) patient had a pelvic recurrence 24 months after treatment,^[6]^ and 1 of 5 (20%) patient had multiple recurrences. The first recurrence was in the pelvis 8 months after treatment, the second recurrence was on the iliac artery 7 months after the first recurrences, and 2 years later recurrences were seen in the liver, vagina, and iliac artery.^[19]^ Median DFS was of 5 months and the mean was 9 months in the FOD-relapsed patients, and OS was not reported. One of 8 (12.5%) patient died after 2 recurrences, the first relapse was on vaginal apex and the second one was on ileum, recurrences occurred 40 and 200 months after treatment, respectively.^[23]^ Two of 8 (16.7%) patients were alive with disease, 1 of 2 (50%) after multiple recurrences, the first recurrence was in abdomen, the second one was on ovary, and the third one was in an unspecified site, recurrences occurred 1, 10, and 12 months after treatment, respectively^[25]^ and 1 of 2 (50%) after a peritoneal recurrence 12 months later and multiple intra-abdominal, pelvic, and peritoneal recurrences surgically treated. For the 5 of 53 (9.4%) DOD patients mean OS was 42.4 months.^[13,25]^

### 4.3 Mitotic count

IMT is characterized by low mitotic activity. The number of mitoses per 10 HPF were reported in 51 of 72 (70.8%) patients. Mean number of mitoses per 10 HPF was 4.6 (range 0–24 mitoses per 10 HPF).

### 4.4 Immunohistochemistry

Immunohistochemistry for ALK has been performed in 62 of 72 (86.1%) patients. ALK overexpression was detected in 58 of 62 (93.5%) patients whilst in 4 of 62 (6.4%) patients was not detected.^[4,7,18,25]^ Desmin expression was tested in 58 of 72 (80.5%) patients, it was positive in 48 of 58 (82.7%) patients. Actin expression was tested in 48 of 72 (66.7%) patients, it was positive in 43 of 48 (89.6%) patients. Cytokeratin expression was tested in 12 of 72 (16.7%) patients, it was positive in 2 of 12 (16.7%) patients. Vimentin expression was tested in 6 of 72 (8.3%) patients, it was positive in 5 of 6 (83.3%) patients. CD34 expression was tested in 8 of 72 (11.1%) patients, it was positive in 1 of 8 (12.5%) patients. CD68 expression was tested in 5 of 72 (6.9%) patients, it was positive in 4 of 5 (80%) patients. H-caldesmon expression was tested in 26 of 72 (36.1%) patients, it was positive in 10 of 26 (38.5%) patients. Interleukin-6 (IL-6) expression was tested in 1 of 72 (1.4%) patient and resulted positive.^[3]^ CD117 expression was tested in 6 of 72 (8.3%) patients and resulted negative in all patients. S100 expression was tested in 15 of 72 (20.8%) patients and resulted negative in all patients. Estrogen receptor (ER) was tested in 22 of 72 (30.5%) patients, it was positive in 14 of 22 (63.6%) patients. Progesterone receptor (ER) was tested in 16 of 72 (22.2%) patients, it was positive in 14 of 16 (87.5%) patients.

### 4.5 Fluorescence in situ hybridization

FISH was performed in 43 of 72 (59.7%) patients, FISH resulted positive in 34 of 43 (79.1%) patients and negative in 6 of 43 (13.9%) patients. In addition, FISH failed in 2 of 43 (4.6%) patients^[7,25]^ because the quality of the extracted RNA was not optimal (excessively degraded RNA) and in 1 of 43 (2.3%) patient FISH showed an abnormal pattern characterized by a single-isolated green 5’ ALK probe (in addition to a single normal unsplit red-green pair).^[25]^

Immunohistochemical staining and FISH were both performed in 42 of 72 (58.3%) patients and resulted both positive in 32 of 42 (76.2%) patients and both negative in 2 of 42 (4.8%) patients.^[4,7]^ Immunohistochemical staining was positive and FISH negative in 4 of 42 (9.5%) patients.^[4,7,13,25]^ In 3 of 42 (7.1%) patients was not possible obtain a clear result by FISH.^[7,25]^

## 5 Discussion

Since 1973, when the first case of IMT was described, 72 cases of uterine IMT have been reported in literature^[3–27]^ (Table 1  ). Sixty-five of 72 (90.3%) IMT arose in the corpus and 7 of 72 (9.7%) arose in the cervix. The mean age of patients with uterine IMT was 40.6 years (range, 6–78 years; SD ± 14.9). The mean age of patients with corpus and cervix IMT patients was similar, 41.0 years (SD ± 15.7) and 39.0 years (SD ± 15.6), respectively. Previous studies report a similar age range in the uterine IMT patients,^[7,26–29]^ instead extrauterine IMT has a predilection for children and adolescents.^[26,27]^ IMT usually presents as a mass and often clinicians presume it is a leiomyoma. Grossly, IMTs may be firm, fleshy, or gelatinous, with a white or tan cut surface. Calcification, hemorrhage, and necrosis are identified in a minority of cases.^[28]^ According to previous studies huge tumors are rare,^[7,28,29]^ our patient had a tumor of 3 cm (Fig. 3), and at literature review, uterine IMT mean size was 6.8 cm (SD ± 4.4 cm). No specific symptoms were reported, the most common were abdominal/pelvic pain and vaginal bleeding, moreover fever/weight loss know as constitutional syndrome was reported in 5 of 35 (14.3%) uterine IMT patients. In a previous review this constitutional syndrome was seen in 15% to 30% of IMT patients^[28]^ and was associated with microcytic anemia, a raised erythrocyte sedimentation rate, thrombocytosis, and/or polyclonal hypergammaglobulinemia.^[15]^ Treatment of uterine IMT was reported for 48 of 72 (66.7%) patients. Surgery was the most common treatment, it was reported for 44 of 48 (91.7%) patients, particularly 23 of 44 (52.3%) patients underwent hysterectomy and 10 of 44 (22.7%) patients underwent tumor resection without hysterectomy. Moreover, 4 of 48 (8.3%) patients received only follow-up because the tumor was unresectable or metastatic.^[20–22,26,27]^ Follow-up information was available for 53 of 72 (73.6%) patients. Forty-six of 53 (86.8%) patients were FOD despite having one or more relapses during follow-up and OS in this group was 29.2 months. Most FOD patients had undergone surgery but the patients who had only received follow-up showed a spontaneous regression of IMT. There was no difference in OS between treated and untreated patients (29.3 and 22 months, respectively). IMT recurrence rate varies by anatomical site, from 2% for IMT confined to the lung to 25% for extrapulmonary IMT. Recurrences are particularly common when IMT is not completely resected as in case of multinodular intra-abdominal IMT and in the case of delicate anatomical locations such as the larynx or trachea. On contrary, recurrence is very infrequent when a solitary IMT is completely resected.^[28]^ In our review 8 of 53 (15.1%) patients had recurrences, particularly, 4 of 8 (50%) patients had multiple recurrences.^[19,25,26,27]^ Recurrences occurred from 1 to 200 months after treatment. Five of 8 (62.5%) were FOD,^[6,19,23]^ 1 of 8 (12.5%) patient died after 2 recurrences 200 months after treatment (23), and 2 of 8 (25%) patients was alive with disease after multiple recurrences.^[25]^ Moreover, 4 of 8 (50%)^[19,23,26,27]^ patients with recurrence had had a delay in diagnosis of IMT. First diagnosis had been of smooth muscle tumor of uncertain malignant potential (STUMP), afterwards the specimens were revised for pathology confirmation because the natural history of rapid recurrences after initial local management was clearly inconsistent with a typical STUMP. Immunostains showed diffuse positivity for ALK1 expression and comprehensive genomic profiling identified an in frame DCTN1-ALK gene fusion. The diagnosis of STUMP was revised to that of an IMT with myxoid features. One of the 2 patients was treated with an ALK inhibitor and a multikinase VEGF inhibitor.^[19]^ In our review, mean DFS was of 12.7 months for the 8 relapsed patients but OS was reported only in 3 cases and the mean is 49.3 months. Distant metastasis of IMT occur in 2% to 5% of cases.^[6,28]^ The primary tumors affected patients over a broad age range (17 months to 79 years) and arose in a variety of anatomical sites. The most common sites of metastasis are lung and brain, followed by liver and bone.^[26–28]^ Metastatic disease is usually identified at presentation or within a year of diagnosis,^[26,27]^ but occasional patients develop metastases up to 9 years following excision.^[30]^ To date, only 2 cases of metastatic uterine IMT have been reported in literature, in this case pelvic lymph nodes and distant metastasis were present already at the time of diagnosis.^[20,26,27]^ Both IMT patients received an initial misdiagnosis of leiomyosarcoma.^[20,26,27]^ Subsequently metastases regressed spontaneously in 1 case,^[20]^ whereas in the second case underwent to both further debulking surgery and tyrosine kinase inhibitor.^[26,27]^ Moreover, 5 of 53 (10.4%) patients DOD. One of 5 (20%) died 27 months after treatment,^[13]^ 1 of 5 (20%) died after 48 months,^[13]^ 1 of 5 (20%) died after a month,^[25]^ 1 of 5 (20%) died after 4 months,^[25]^ and 1 of 5 (20%) died after 132 months and 2 recurrences. The mean OS for these patients was 42.4 months. These IMT patients presented an aggressive disease that caused a misdiagnosis of myxoid leiomyosarcoma of the uterus. After their death, tumor specimens were tested for ALK positivity and IMT diagnosis was done.^[13]^

In previous studies, ALK positivity was reported in 100% and 87.5% of uterine IMT,^[4,6,8]^ stronger ALK expression was found in myxoid areas compared with fascicular bundles.^[6]^ In our review, ALK expression was tested in 62 of 72 (86.1%) patients. ALK overexpression was detected in 58 of 62 (93.5%) patients.^[3,4,6–8,10,12,13,20,24–27]^ ALK expression in female genital tract IMT appears to be frequent, especially when compared with other anatomic sites but ALK negative IMT may occur in the female genital tract as well,^[6]^ particularly in adults.^[8]^ As expected, given their myofibroblastic differentiation, IMT in our patient was positive for actin, IMTs are generally positive for smooth muscle markers such as actin (in 43/48, 89.6% patients), desmin (48/58, 87.7% patients), and vimentin (5/6, 83.3% patients) (Table 1  ). IL-6 expression was tested in 1/72 (1.4%) patient and resulted positive.^[3]^ IL-6 overexpression and high serum level of IL-6 were associated with inflammatory constitutional symptoms such as fever, weight loss, fatigue, and a variety of laboratory abnormalities, such as acute-phase reaction, thrombocytosis, anemia, and elevated sedimentation ratio. The disappearance of constitutional symptoms and the fall in serum IL-6 level were obtained after IMT excision.^[3]^ Moreover, no clear prognostic factors are identified for IMT.^[21]^ Tumor size, tumor necrosis, cellularity, mitotic activity do not appear to be correlated with outcome.^[21]^ On contrast, in our review highest mitotic count per 10 HPF were reported in a patient who relapsed 24 months after treatment (20 mitoses per 10 HPF)^[6]^ and in a patient who died 12 months after treatment (24 mitoses per 10 HPF).^[25]^ Moreover, tumor cell necrosis, larger tumor size, higher mitotic activity, a predominantly myxoid pattern, and infiltrative borders were seen only in cases with recurrence or metastasis.^[6]^ The pattern of infiltration in these cases varied, appearing as a continuous, markedly irregular (geographic) interface, or as discontinuous finger-like projections into the surrounding myometrium.^[6]^ Furthermore, the presence of an epithelioid or round cell morphology and a distinct perinuclear or nuclear membrane ALK immunohistochemical staining has been described in aggressive intra-abdominal IMTs named “epithelioid inflammatory myofibroblastic sarcoma.”^[11,30]^ However, ALK positivity represents the most important test to suspect an uterine IMT.

Uterine IMT is a rare tumor, that can be misdiagnosed with smooth muscle tumors with myxoid differentiation such as myxoid leiomyosarcoma,^[13]^ STUMP,^[19]^ myxoid leiomyoma,^[7]^ and atypical leiomyoma.^[26,27]^ In the uterus, expression of ALK in IMT mimics, has not been reported.^[29]^ Therefore ALK immunohistochemistry should be performed in all cases that morphologically raise the possibility of an IMT (myxoid ± fascicular growth, lymphoplasmacytic infiltrate, fasciitis-like appearance of tumor cells), and, if positive, regardless of the intensity, confirmation by FISH should be considered.^[6,29]^ In our patient, neoplastic cells showed ALK rearrangement in 91% of cell nuclei. In our review, immunohistochemical staining and FISH were both performed in 42 of 72 (58.3%) patients and resulted both positive in 32 of 42 (76.2%) patients and both negative in 2 of 42 (4.8%) patients.^[4,7]^ Immunohistochemical staining was positive and FISH negative in 4 of 42 (9.5%) patients.^[4,7,13,25]^ Knowing that ALK is overexpressed is of great importance because new targeted therapies using tyrosine kinase inhibitor might be used.^[19,26,27,31,32]^

In the last years the interest regarding IMT is progressively increased between pathologists but yet few cases has been reported in literature. Nevertheless, we think that gynecologists should know this tumor and how to manage it because probably it is more common than it was believed. They should know that the diagnosis of IMT should be particularly considered in pregnancy or in case of submucosal/ polypoid tumor.

A diagnostic hysteroscopy should be performed in every case of intrauterine polypoid mass, a biopsy can guide the right management that in case of IMT could be just hysteroscopy excision. On contrary, curettage should not be considered as exclusive treatment because unable to completely remove IMT.

Comparing treatment and outcome according to preoperative diagnosis of IMT we found that 23 of 48 (47.9%) patients^[3,6–9,16,26,27]^ had IMT diagnosis after that hysterectomy had been performed for other indications, of these 23 patients, 18 of 23 (78.3%) were FOD,^[3,6–9,16,26,27]^ 2 of 23 (8.7%) patients relapsed^[8]^ and in 3 of 23 (13.0%) patients follow-up data were not reported.^[6,8]^ Five of 48 (10.4%) patients received hysterectomy after that a biopsy of tumor had been performed, 4 of 5 (80%) patients had a diagnosis of IMT^[9,14,18,20]^ at biopsy and 1 of the 5 (20%) patients had a misdiagnosis of leiomyosarcoma^[12]^ at biopsy. All 5 patients were FOD. Moreover, 20 of 48 (41.7%) patients did not received hysterectomy after that a diagnosis of IMT^[8,10,21,22,24,26,27]^ or misdiagnosis of leiomyoma,^[6,7,26,27]^ STUMP^[19,23]^ had been done at biopsies. Particularly, 15 of 20 (75%) not hysterectomized patients were FOD, 5 of 20 (25%) not hysterectomized patients relapsed.^[6,19,23,26,27]^ Two of 5 (40%) relapsed patients were treated with ALK inhibitor and were FOD^[19,23]^; 1 of 5 (20%) relapsed patient was surgically and hormonally treated and was AWD^[26,27]^ and in 2 of 5 (40%) relapsed patients follow-up data were not reported.^[6,26,27]^

It should be underlined that 75% of IMT patients that did not receive hysterectomy after preoperative diagnosis were FOD and that in case of IMT relapse an effective rescue therapy with ALK inhibitor could increase the number of FOD patients.^[19,23]^ Hence, we advocate that young patients that want to have pregnancy might delay hysterectomy at the end of childbearing age, on contrary women that do not desire pregnancy should be treated with hysterectomy to avoid a low but demonstrated risk of relapse.

Surgical treatment, particularly complete surgical resection, seems to represent the best treatment for IMT. Mini invasive surgery should be chosen to diagnose and to treat uterine IMT, both hysteroscopy^[8–10]^ and laparoscopy seem to be effective and safe. However, IMT morcellation should be avoided because the risk of addominal/pelvic recurrence.^[19,23,26,27]^ Considering that the age of the first pregnancy increases progressively^[32]^ and that uterine IMT arise in young women, in case of desire of offspring, in case of unresectable primary^[21]^ or metastatic IMT,^[20]^ a “watch and wait” strategy might be safe to avoid an aggressive surgery and to preserve fertility. Interestingly, a strong association seems to be between IMT and pregnancy.^[24,26,27]^ Rarely, IMT can recur or even be lethal, in these cases if surgery is not feasible a successful targeted therapy using tyrosine kinase inhibitor therapies could represent a valuable alternative.
